# Supplementary material for: Prediction of etiology and prognosis based on hematoma location of spontaneous intracerebral hemorrhage: a multicenter diagnostic study
Source: Neuroradiology. 2025 Jun 3;67(7):1761–72. doi: 10.1007/s00234-025-03661-7 (PMC12390878; doi:10.1007/s00234-025-03661-7)
Supplement: Supplementary file 1 — Supplementary Material 1 [file 234_2025_3661_MOESM1_ESM.pdf]

Supplementary Materials and Methods

Prediction of etiology and prognosis based on hematoma location of spontaneous intracerebral hemorrhage: a multicenter diagnostic study

Supplementary Methods

Study Population enrollment

Inclusion and exclusion interflow are shown in Supplementary Figure 1.

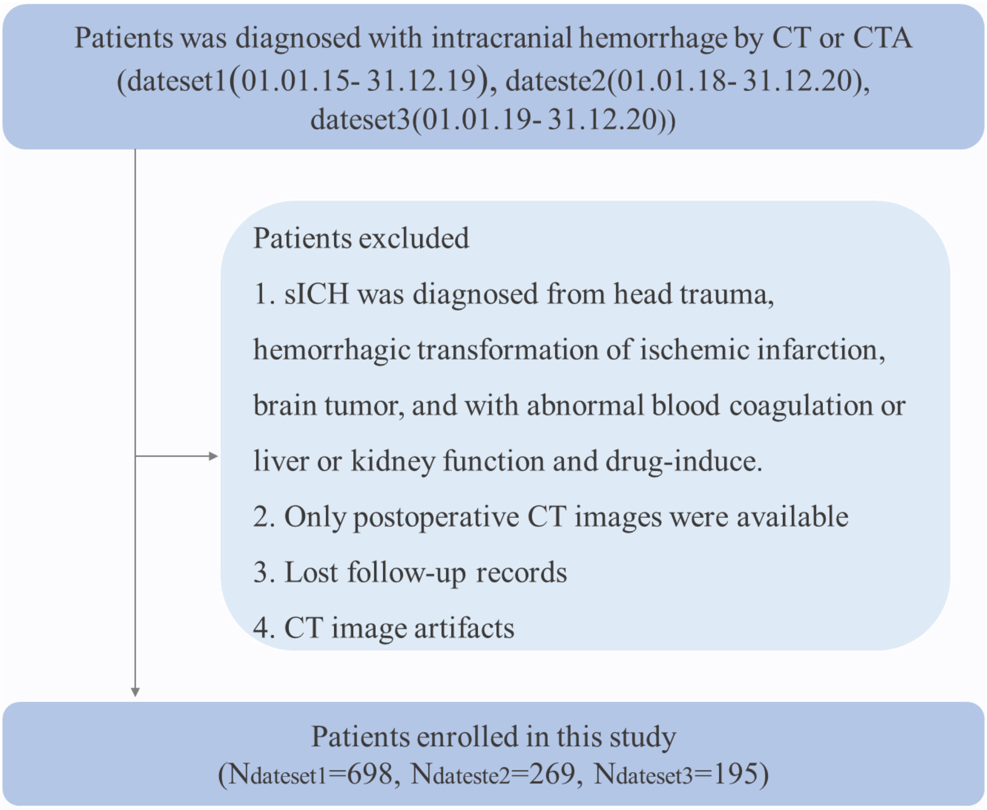

Supplementary Figure 1. Flowchart shows the patient enrollment process.

dateset1 = Jinling Hospital, dateset2= Yi Jishan Hospital, dateset3 = Nanjing First Hospital

sICH= Spontaneous cerebral hemorrhage; CTA= Computer tomography angiography.

Supplementary Table1. Intracerebral regions in the parenchyma, subarachnoid space, and ventricles.

JHU template		in-house template	
**Abbreviation**	**Full title**	**Abbreviation**	**Full title**
Ins	insular	AFC	anterior longitudinal fissure cistern
Amyg	amygdala	PFC	posterior fissure cistern
Hippo	hippocampus	LFC	lateral fissure cistern
Caud	caudate nucleus	SC	suprasellar cistern
Put	putamen	AC	ambient cistern
GP	globus pallidus	PC	pontine cistern
Thal	thalamus	QC	quadrigeminal cistern
Hypothalamus	hypothalamust	Ventricle_L	left lateral ventricle
Mynert	nucleus innominata of mynert l	Ventricle_R	Right lateral ventricle
NucAccumbens	nucleus accumbens	Ventricle_T	third ventricle
RedNc	red nucleus	Ventricle_F	fourth ventricle
Snigra	substancia nigra		
cerebellum	cerebellum		
CP	cerebral peduncle		
Midbrain	midbrain		
PIns	posterior insula		
CST	corticospinal tract		
SCP	superior cerebellar peduncle		
MCP	middle cerebellar peduncle		
PCT	pontine crossing tract		
ICP	inferior cerebellar peduncle		
ML	medial lemniscus		
Pons	pons		
Medulla	medulla		
ACR	anterior corona radiata		
SCR	superior corona radiata		
PCR	posterior corona radiata		
GCC	genu of corpus callosum		
BCC	body of corpus callosum		
SCC	splenium of corpus callosum		
TAP	tapatum		
ALIC	anterior limb of internal capsule		
PLIC	posterior limb of internal capsule		
RLIC	retrolenticular part of internal capsule		
EC	external capsule		
CGC	cingulum (cingulate gyrus)		
CGH	cingulum (hippocampus)		
Fx/ST	fornix (cres) / Stria terminalis		
Fx	Fornix		

IFO	Inferior fronto-occipital fasciculus
PTR	Posterior thalamic radiation
SS	Sagittal stratum
SFO	Superior fronto-occipital fasciculus
SLF	Superior longitudinal fasciculus
UNC	Uncinate fasciculus
AnsaLenticularis	Ansa lenticularis
AnteriorCom	Anterior commissure
LenticularFasc	Lenticular fasciclus
OlfactoryRadiation	olfactory radiation
Mammillary	mammillary body
OpticTract	optic tract
SFG	superior frontal gyrus
MFG	middle frontal gyrus
IFG	inferior frontal gyrus pars triangularis
LFOG	lateral fronto-orbital gyrus
MFOG	middle fronto-orbital gyrus
RG	gyrus rectus
PoCG	postcentral gyrus
PrCG	precentral gyrus
SPG	superior parietal gyrus
SMG	supramarginal gyrus
AG	angular gyrus
PrCu	pre-cuneus
STG	pole of superior temporal gyrus
MTG	pole of middle temporal gyrus
ITG	inferior temporal gyrus
PHG	parahippocampal gyrus
ENT	entorhinal area
FuG	fusiform gyrus
SOG	superior occipital gyrus
MOG	MIDDLE OCCIPITAL GYRUS
IOG	inferior occipital gyrus right
Cu	cuneus
LG	lingual gyrus
rostral	rostral anterior cingulate gyrus
subcallosal	subcallosal anterior cingulate gyrus
subgenual	subgenual anterior cingulate gyrus
dorsal	dorsal anterior cingulate gyrus
PCC	posterior cingulate gyrus

PSTG	posterior superior temporal gyrus
PSMG	posterior middle temporal gyrus
PSIG	posterior inferior temporal gyrus

Radiomics features extraction

We used the python package PyRadiomics (Version 3.0.1; {https://pypi.org/project/pyradiomics}). For the patient level radiomic feature H, we define a bag $H = \{\overrightarrow{h_1}, \cdots \overrightarrow{h_\iota}, \cdots \overrightarrow{h_K}\}$, where K is the number of lesions in a patient (K is an integer and K≤5) and $\overrightarrow{h_\iota}$ denotes the radiomic feature vector of the i-th lesion called instances. $\overrightarrow{h_\iota} = \{f_{i,1}, \ldots, f_{i,j,} \ldots, f_{i,1454}\}$. The patient-level radiomic feature H is calculated by weighted summation of radiomic features of all delineated lesions as depicted in Equation 1.

The adaptive weight $a_i = \{a_{i,1}, a_{i,2}, \ldots, a_{i,j}, \ldots a_{i,1454}\}$ was constructed using a deep learning multi-sample attention model algorithm (Equation 2), where the w and V are network parameters with dimensions of $K \times 1454$. The sum of a_i for each radiomic feature in an individual patient is 1. After adaptive weighting, the radiomic features (***H***) of a single case was obtained, with the dimension of 1454×1.The training process was conducted in the training cohort with a five-fold cross validation strategy. Specifically, the dataset was divided into five folds, where four-folds were used for training and one-fold for validation. The process was repeated five times to ensure every single fold had been used as a validation set once. In each iteration, the radiomic features were extracted from multiple lesions and all 1454 radiomic features were combined as a vector for each lesion. Then, the attention algorithm was applied as described in the previous two steps to generate a patient-level feature set ***H***. It was then fed to a multiple layer perception classifier to generate a fully connected layer with the anticipated bag-level label, sICH was the binary clinical outcome in our study (good prognosis and bad prognosis) and the errors (i.e. function loss) were used to adjust the

values of the parameters until a minimized error was reached. The preliminary w and V were determined in the training process and were tuned in the validation fold afterwards.

After five iterations, the mean w and V were used as the final parameters and were kept constant for the rest of the study. Consequently, the adaptive weight coefficients for all lesions and all cases were computed using Equation 2. The patient-level radiomic features were constructed (Supplementary Figure 2) .

Finally, the Lasso feature selection method was applied to select the features with non-zero coefficients from all the weighted features, and 47 features were retained in the end.

$$H = \sum_{i=1}^{n=K} a_i \cdot \overrightarrow{h_i} = \left[\sum_{i=1}^{n=K} a_{i,1} h_{i,1}, \quad \dots, \quad \sum_{i=1}^{n=K} a_{i,j} h_{i,j}, \quad \dots, \quad \sum_{i=1}^{n=K} a_{i,1454} h_{i,1454}\right] (1)$$

$$a_i = \frac{\exp\left\{w_i^T \tanh\left(V_i \overrightarrow{h_i}^T\right)\right\}}{\sum_{l=1}^{K} \exp\{w_i^T \tanh(V_i \overrightarrow{h_i}^T)\}} \text{, where } w_i \epsilon R^{1\times1454} \text{ and } V_i \epsilon R^{1\times1454} \quad (2)$$

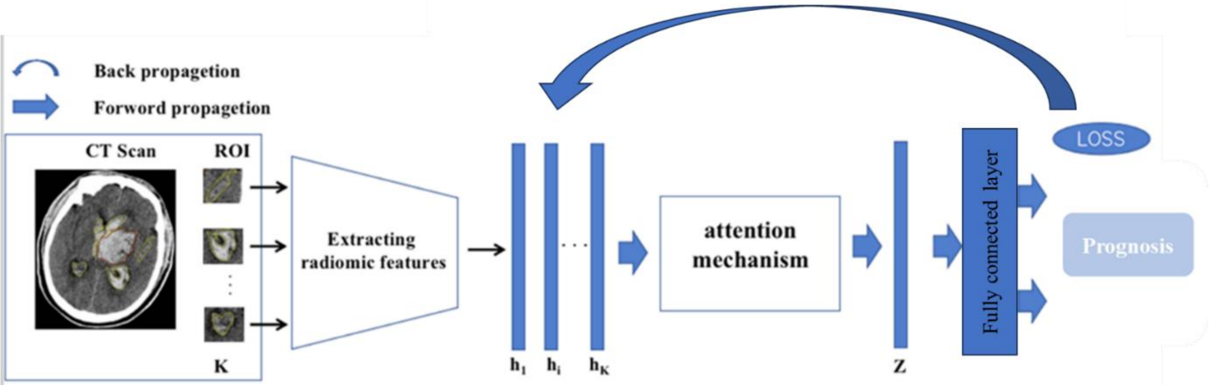

Supplementary Figure 2. Attention mechanism-weighted radiomics feature map

Supplementary Results

Clinical characteristics

Clinical characteristics of development cohort (dataset 1) and external-validation cohorts (dataset 2 and 3) are presented in Supplemental Table1 and 2. There were statistically significant differences in gender and etiology among the three datasets (all $p<0.05$), statistically significant differences in gender and prognosis between datasets 1 and 2, but no statistically significant differences in gender and prognosis in dataset 3 ($p>0.05$). Stroke history and etiology were statistically significant in the all datasets (all $p<0.05$), stroke history and prognosis were statistically significant in data sets 1 and 3 (all $p<0.05$). Smoking and etiology were statistically significant in dataset 1 ($p<0.05$), but were not statistically significant in etiology and prognosis in the other two datasets (all $p>0.05$). Diabetes mellitus and etiology and prognosis were statistically significant in data sets 1 and 3 (all $p<0.05$), and statistically significant in dataset 2 (all $p<0.05$), but not statistically significant in prognosis (all $p>0.05$). The time from onset to baseline CT and etiology were statistically significant in the three datasets (all $p<0.05$), but there was no statistical significance between the data sets and the prognosis (all $p<0.05$). There were no significant differences in the etiology and prognosis of alcohol consumption and hyperlipidemia among the three datasets (all $p>0.05$) (Supplementary Table 1, 2).

Supplementary Table2. Demographic, Clinical, and Baseline Variables of the Study Population for etiological prediction

**Variables**	**Dateset1**				**Dateset2**				**Dateset3**			
	**Hypertension (n=337)**	**Aneurysm (n=276)**	**Vascular malformation (n=58)**	***p* value**	**Hypertension (n= 138)**	**Aneurysm (n= 82)**	**Vascular malformation （n=30）**	***p* value**	**Hypertension (n= 88)**	**Aneurysm (n= 77)**	**Vascular malformation （n=23）**	***p* value**
Age, median (IQR)	57 (49-66)	54 (47-63)	46 (30.75-57.25)	<0.001*	62 (55-71)	62 (55-69)	48 (37.5-54.75)	<0.001*	64.5 (54-74)	61 (51-68)	55 (46-59)	0.001*
GCS, median (IQR)	12 (10-13)	14 (10-14)	12 (8.5-14)	<0.001*	11 (8-12)	13 (10-15)	12 (10-14)	<0.001*	12 (10-13)	13 (12-14)	13 (11-13)	<0.001*
Sex, n												
Male	225 (66.8%)	103 (37.3%)	34 (58.6%)	<0.001*	98 (71.5%)	25 (30.5%)	16 (53.3%)	<0.001*	70 (79.5%)	33 (42.9%)	14 (60.9%)	<0.001*
Female	112 (33.2%)	173 (62.7%)	24 (41.4%)		40 (29.0%)	57 (69.5%)	14 (46.7%)		18 (20.5%)	44 (57.1%)	9 (39.1%)	
Hypertension, n	337 (100.0%)	108 (39.1%)	12 (20.7%)	<0.001*	137 (100.0%)	53 (64.6%)	9 (30.0%)	<0.001*	88 (100.0%)	43 (55.8%)	7 (30.4%)	<0.001*
Diabetes mellitus, n	48 (14.2%)	12 (4.3%)	3 (5.2%)	<0.001*	16 (11.6%)	2 (2.4%)	1 (3.3%)	0.030*	18 (20.5%)	5 (6.5%)	1 (4.3%)	0.012*
Hyperlipidemia, n	17 (5.0%)	1 (0.4%)	0 (0.0%)	0.001*	3 (2.2%)	1 (1.2%)	0 (0.0%)	0.653	3 (3.4%)	1 (1.3%)	1 (4.3%)	0.608
Smoking, n	72 (21.4%)	33 (12.0%)	6 (10.3%)	0.003*	32 (23.4%)	15 (18.3%)	7 (23.3%)	0.674	23 (26.1%)	14 (18.2%)	7 (30.4%)	0.338
Drinking, n	67 (19.9%)	48 (17.4%)	16 (27.6%)	0.199	26 (19.0%)	11 (13.6%)	5 (16.7%)	0.525	19 (21.6%)	13 (16.9%)	4 (17.4%)	0.726
Onset-to-CT time, n												
Stage1 (<24h)	79 (23.4%)	88 (31.9%)	20 (34.5%)		114(82.6%)	73 (89.0%)	26(86.7%)		78 (88.6%)	66 (85.7%)	20 (87%)	
Stage2 (24h-72h)	186 (55.2%)	146 (52.9%)	26 (44.8%)	0.014*	16(11.6%)	9(11.0%)	2(6.7%)	<0.001*	10 (11.4%)	11 (14.3%)	1 (4.3%)	<0.001*
Stage3	57 (16.9%)	41 (14.9%)	10 (17.2%)		6 (4.3%)	0(0.0%)	2(6.7%)		0 (0.0%)	0 (0.0%)	1 (4.3%)	

(3-7d)												
Stage (>7d)	15 (4.5%)	1 (0.4%)	2 (3.4%)		2(1.4%)	0 (0.0%)	0(0.0%)		0 (0.0%)	0 (0.0%)	1 (4.3%)	
History of stroke, n	29 (8.6%)	2 (0.7%)	2 (3.4%)	<0.001*	31 (22.5%)	6 (7.3%)	3 (10.0%)	0.008*	11 (12.5%)	1 (1.3%)	7 (30.4%)	<0.001*

IQR= interquartile range; GCS= Glasgow Coma Scale; *Significant difference (P<0.05)

Supplementary Table3. Demographic, Clinical, and Baseline Variables of the Study Population for prognosis prediction

Variables	Dateset1			Dateset2			Dateset3		
	mRS<3 (n=448)	mRS>=3 (n=250)	*p* value	mRS<3 (n= 163)	mRS>=3 （n=106）	*p value*	mRS<3 (n= 110)	mRS>=3 （n=85）	*p value*
Age, median (IQR)	52 (46-61.25)	61.5 (52 -69)	<0.001*	59 (53-70)	59 (53-70)	<0.001*	55.5 (50-64.75)	68 (58-75)	<0.001*
GCS, median (IQR)	14 (11.75-14)	10 (8-12)	<0.001*	13 (11-14)	10 (7.25-12)	0.008	13 (13-14)	11 (9-12)	<0.001*
Sex, n									
Male	218(48.7%)	101(40.4%)	0.036	85 (52.1%)	69 (65.1%)	0.036	65 (59.1%)	57 (67.1%)	0.254
Female	230(51.3%)	149(59.6%)		78 (47.9%)	37 (34.9%)		45 (40.9%)	28 (32.9%)	
Hypertension, n	243(54.2%)	185(74.0%)	<0.001*	109(66.9%)	85 (80.2%)	0.015	64 (58.2%)	71 (83.5%)	<0.001*
Diabetes mellitus, n	29 (6.5%)	36 (14.4%)	<0.001*	13 (8.0%)	7 (6.6%)	0.675	8 (7.3%)	17 (20.0%)	0.008
Hyperlipidemia, n	9 (2.0%)	9 (3.6%)	0.204	3 (1.8%)	1 (0.9%)	0.937	3 (2.7%)	3 (2.7%)	1
Smoking, n	68 (15.2%)	46 (18.4%)	0.27	30 (18.4%)	30 (28.3%)	0.057	27 (24.5%)	19 (22.4%)	0.721
Drinking, n	78 (17.4%)	56 (22.4%)	0.109	23 (14.2%)	24 (22.6%)	0.076	22 (20.0%)	16 (18.8%)	0.837
Pathogenesis, n									
Hypertension	166(37.1%)	171(68.4%)	<0.001*	71 (43.6%)	66 (62.3%)	0.007	35 (31.8%)	53 (62.4%)	<0.001*
Arterial aneurysm	219(48.9%)	57 (22.8%)		59 (36.2%)	23 (21.7%)		55 (50.0%)	22 (25.9%)	
Vascular malformation	36 (8.0%)	22 (8.8%)		17 (10.4%)	13 (12.3%)		17 (15.5%)	6 (7.1%)	
Unknown	27 (6.0%)	0 (0.0%)		16 (9.8%)	4 (3.8%)		3 (2.7%)	4 (4.7%)	
Onset-to-CT time, n									
Stage1 (<24h)	116	79	0.263	126	92	0.155	92(83.6%)	78 (91.8%)	0.262

Stage2 (24h-72h)	242	371		22	12		16 (14.5%)	7 (8.2%)	
Stage3 (3-7d)	79	113		12	2		1 (0.9%)	0 (0.0%)	
Stage (>7d)	11	19		3	0		1 (0.9%)	0 (0.0%)	
History of stroke, n	13 (2.9%)	21 (8.4%)	<0.001*	19 (11.7%)	21 (19.8%)	0.066	7 (6.4%)	12 (14.1%)	0.07

IQR= interquartile range; GCS= Glasgow Coma Scale; mRS= the modified Rankin Scale; *Significant difference (p<0.05

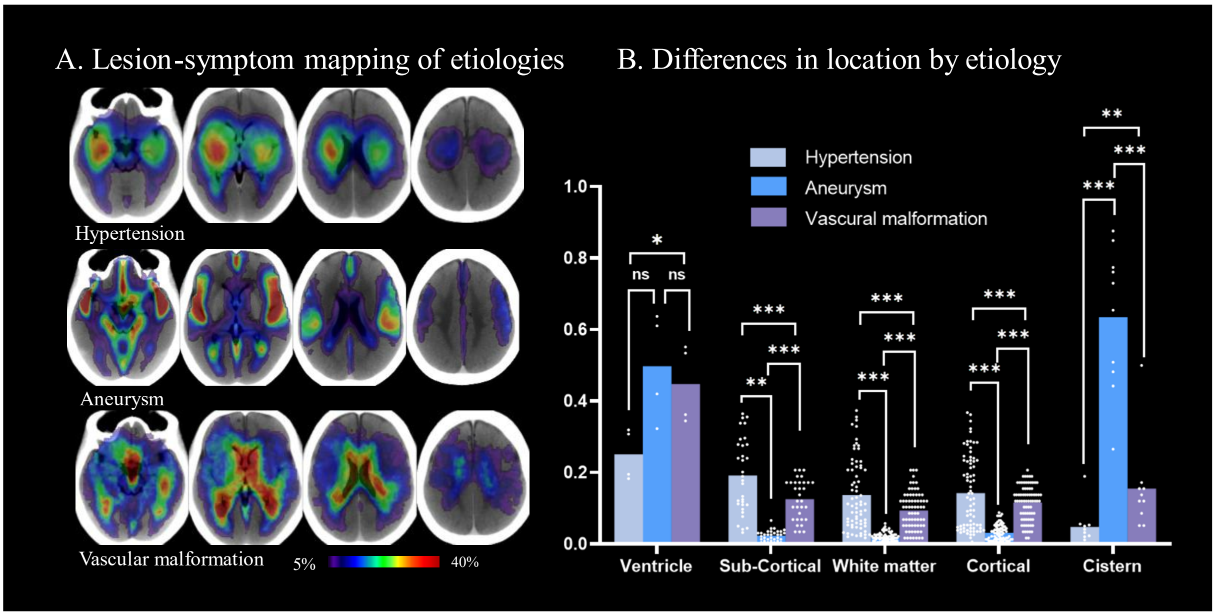

Supplementary Figure 3. Association between hematoma location and etiology in the dataset1 (n = 671). (A) Probability maps depicting the distribution of hematoma location in patients. The location patterns associated with hypertension, aneurysm, and vascular malformation are illustrated, with the frequency of hemorrhage location graded according to color. The gradient scale ranges from 5% to 40%. (B) Paired sample ANOVA was used to compare the involvement rate of each region in each intracranial structure. *: $p < 0.05$, **: $p < 0.01$, ***: $p < 0.001$, ns: $p > 0.05$.

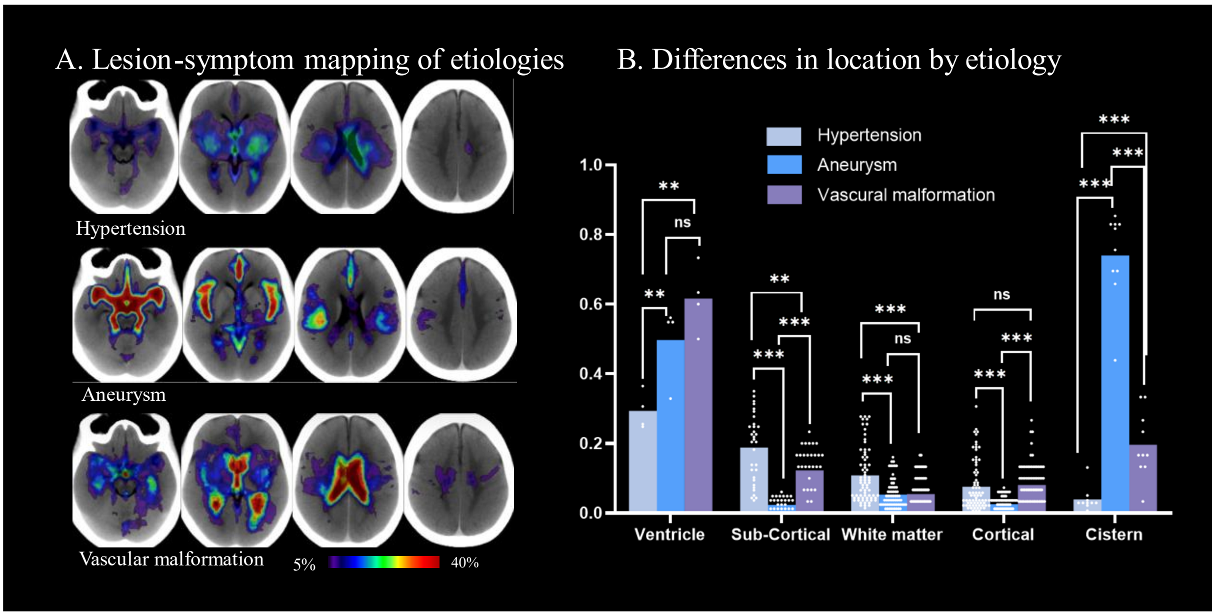

Supplementary Figure 4. Association between hemorrhage location and etiology in the dateset2 (n = 250). (A) Probability maps depicting the distribution of hematoma location in patients. The location patterns associated with hypertension, aneurysm, and vascular malformation are illustrated, with the frequency of hemorrhage location graded according to color. The gradient scale ranges from 5% to 40%. (B) Paired sample ANOVA was used to compare the involvement rate of each region in each intracranial structure. *: $p < 0.05$, **: $p < 0.01$, ***: $p < 0.001$, ns: $p > 0.05$.

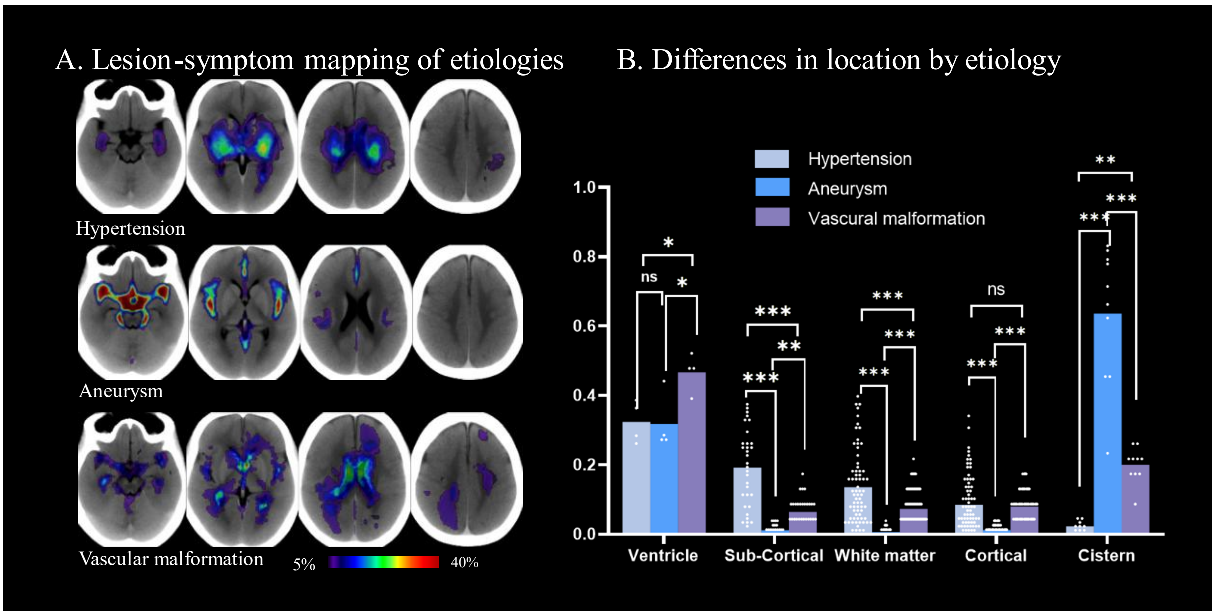

Supplementary Figure 5. Lesion-symptom mapping of hemorrhage location in the dateset3 (n = 188). (A) Probability maps depicting the distribution of hematoma location in patients. The location patterns associated with hypertension, aneurysm, and vascular malformation are illustrated, with the frequency of hemorrhage location graded according to color. The gradient scale ranges from 5% to 40%. (B) Paired sample ANOVA was used to compare the involvement rate of each region in each intracranial structure. *: $p < 0.05$, **: $p < 0.01$, ***: $p < 0.001$, ns: $p > 0.05$.

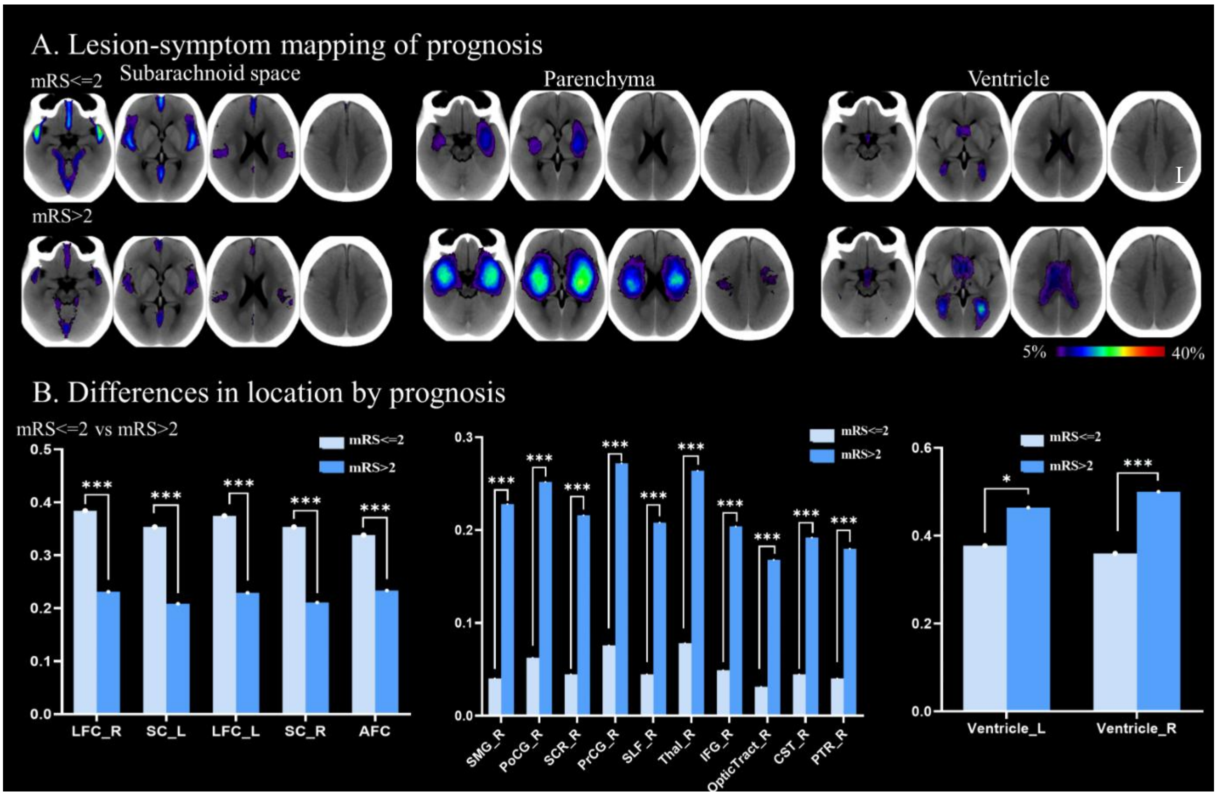

Supplementary Figure 6. Association between hemorrhage location and prognosis in dataset1 (n = 698). (A) Probability maps depicting the distribution of hematoma location in different prognosis. The location patterns associated with subarachnoid space, parenchyma and ventricle are illustrated, with the frequency of hematoma location graded according to color. The gradient scale ranges from 5% to 40%. (B) Frequency plots of the top five cisterns, ten parenchyma and ventricles with group difference, ranked according to chi-square values. *: $p < 0.05$, **: $p < 0.01$, ***: $p < 0.001$. Abbreviations of intracranial region see Supplementary Table 1.

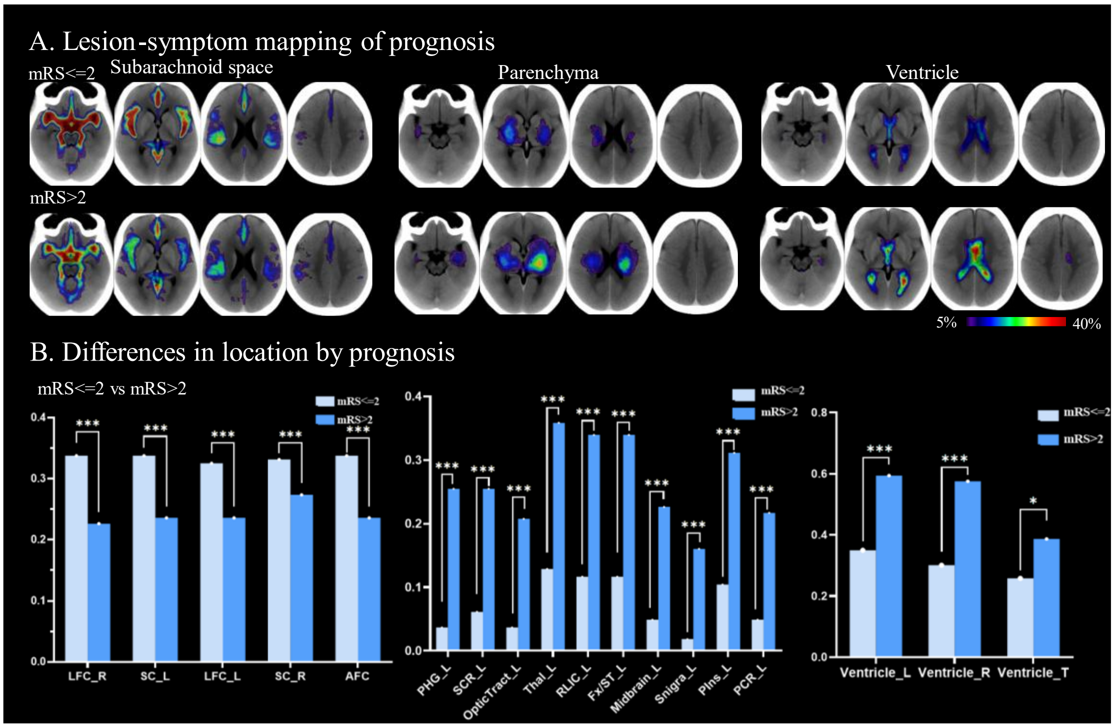

Supplementary Figure 7. Association between hemorrhage location and prognosis in dataset2 (n = 269). (A) Probability maps depicting the distribution of hematoma location in different prognosis. The location patterns associated with subarachnoid space, parenchyma and ventricle are illustrated, with the frequency of hematoma location graded according to color. The gradient scale ranges from 5% to 40%. (B) Frequency plots of the top five cisterns, ten parenchyma and ventricles with group difference, ranked according to chi-square values. *: p < 0.05, **: p < 0.01, ***: p < 0.001. Abbreviations of intracranial region see Supplementary Table 1.

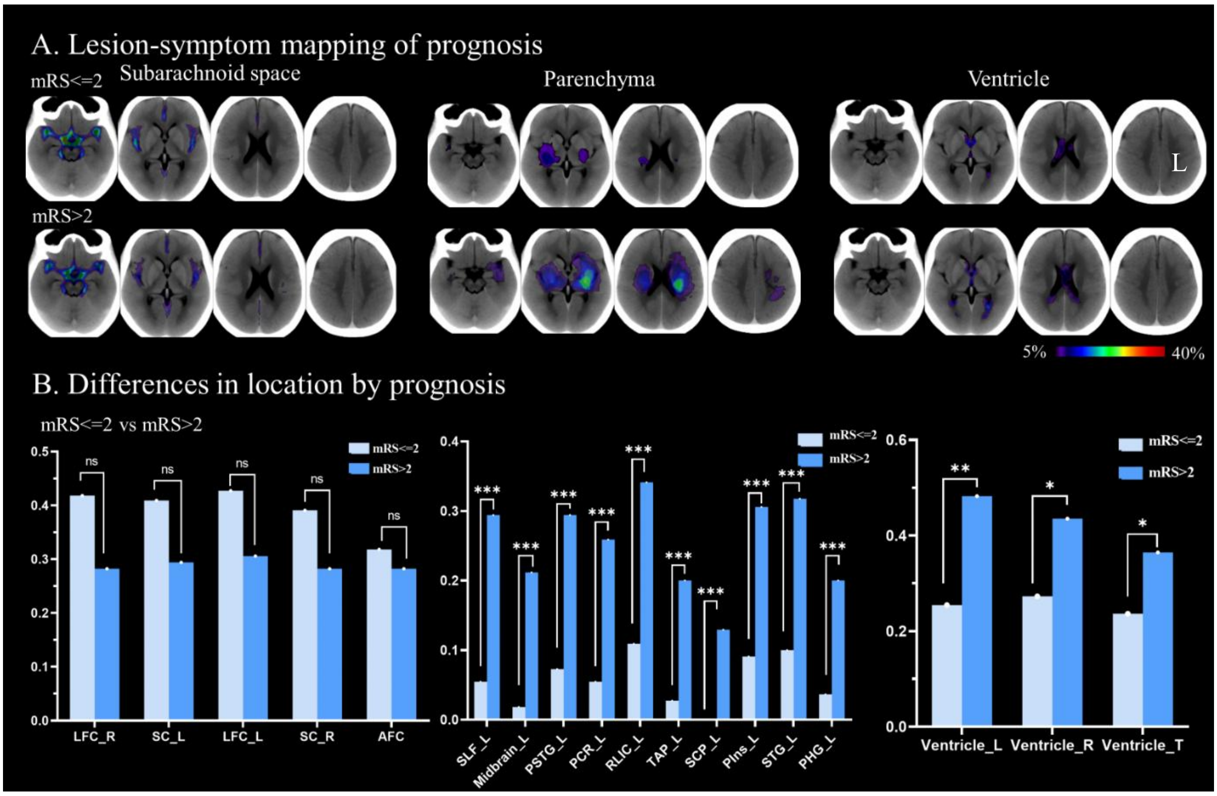

Supplementary Figure 8. Association between hemorrhage location and prognosis in dataset3 (n = 195). (A) Probability maps depicting the distribution of hematoma location in different prognosis. The location patterns associated with subarachnoid space, parenchyma and ventricle are illustrated, with the frequency of hematoma location graded according to color. The gradient scale ranges from 5% to 40%. **(B)** Frequency plots of the top five cisterns, ten parenchyma and ventricles with group difference, ranked according to chi-square values. *: $p < 0.05$, **: $p < 0.01$, ***: $p < 0.001$, ns: $p > 0.05$. Abbreviations of intracranial region see Supplementary Table 1.

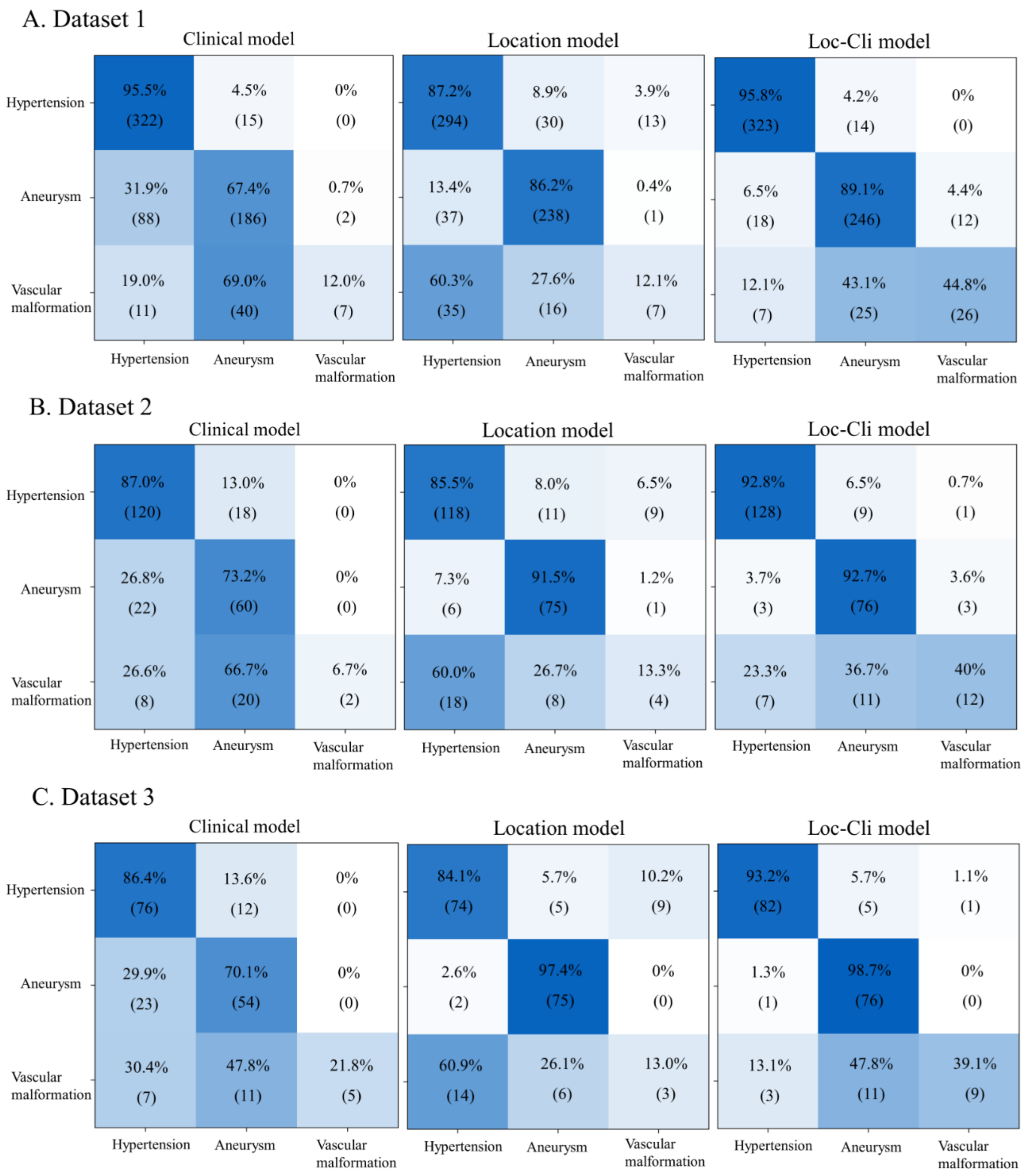

Supplementary Figure 9. The confusion matrix of etiology models. (A-C) The confusion matrix demonstrates the classification capability of the clinical, location and loc-cli models for predicting etiology in dataset 1,2,3. where columns are predicted classes and rows are ground truth.

Loc-Cli= location-clinical

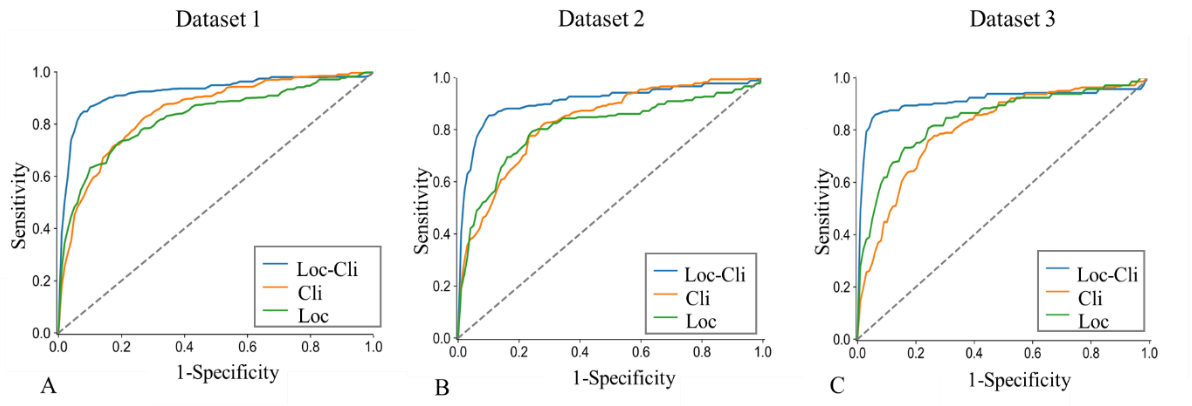

Supplementary Figure 10. Etiology of the ROC curves. ROC curves of etiology models of each dataset.

ROC= Receiver Operating Characteristic

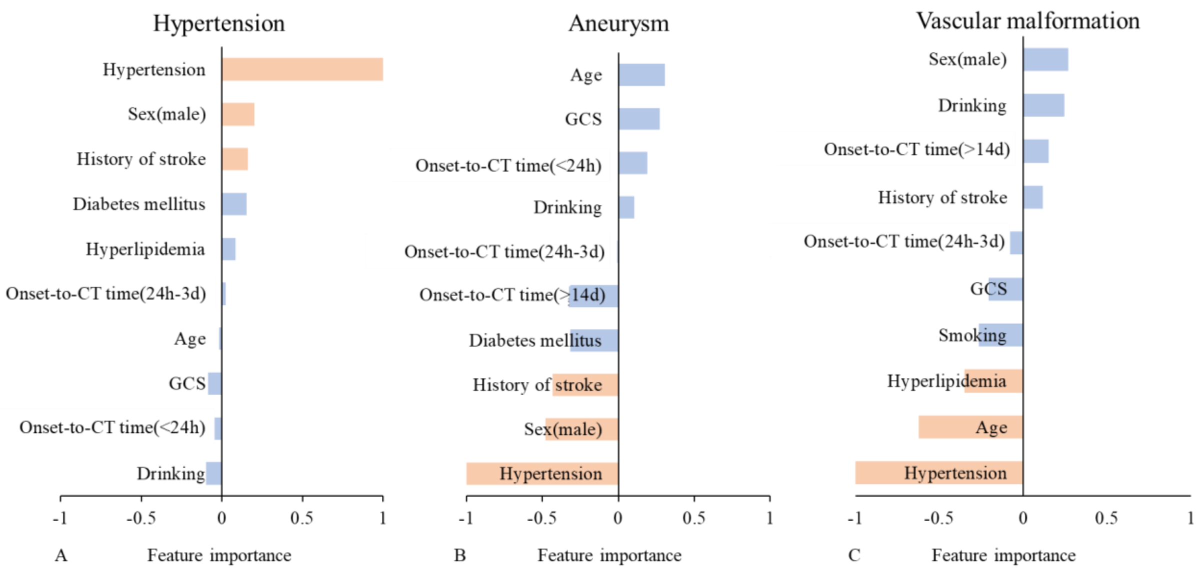

Supplementary Figure 11. **Feature importance of etiology clinical models.** (A-C) Show the top ten clinical features most associated with hypertension, aneurysm, vascular malformation, red represents the three features that contribute the most. Positive values are positively contribution and negative values are negatively contribution. GCS= Glasgow Coma Scale;

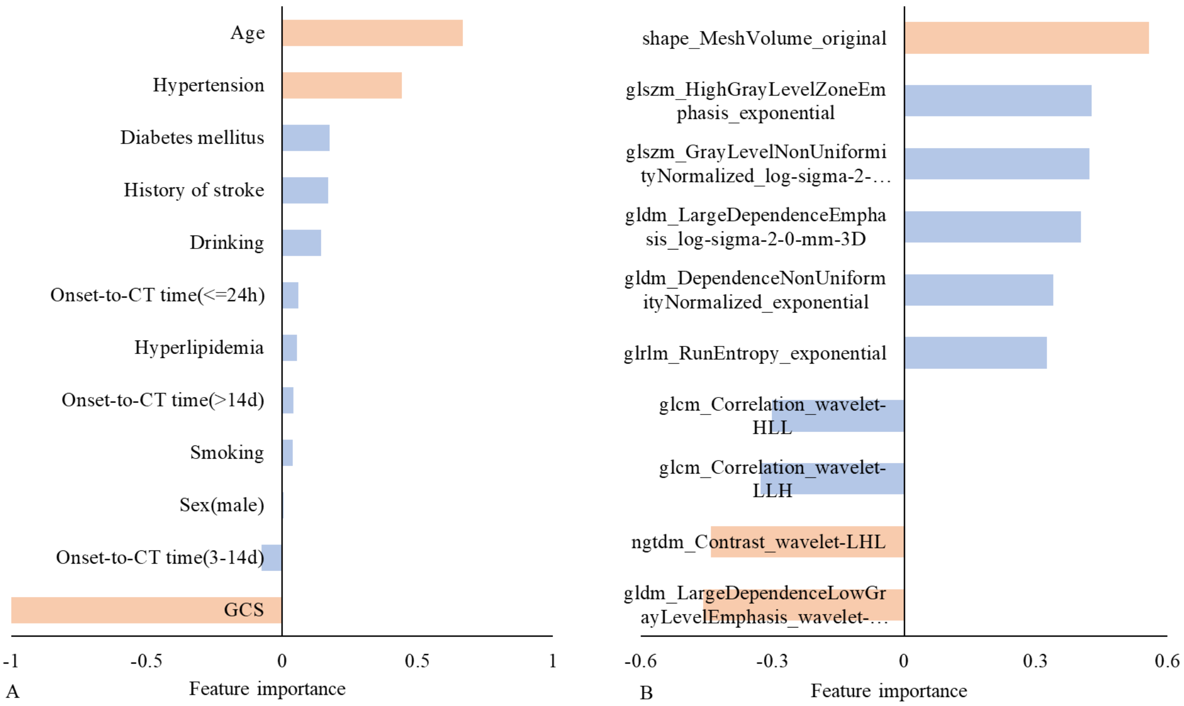

Supplementary Figure 12. Feature importance of prognosis models. (A) The top ten clinical features most associated with prognosis. (B) The top ten radiomics features most associated with prognosis, red represents the three features that contribute the most. Positive values are positively contribution and negative values are negatively contribution.

GCS= Glasgow Coma Scale;

Supplementary Table 4. Performance of clinical model, location model, radiomics model and fusion model prediction task model of all datasets

	Dateset1				Dateset2				Dateset3			
	AUC (95% CI)	Accuracies	Specificity	Sensitivity	AUC (95% CI)	Accuracies	Specificity	Sensitivity	AUC (95% CI)	Accuracies	Specificity	Sensitivity
**Etiology**												
Clinical model	0.848 (0.828-0.867)	0.767	0.858	0.583	0.831 (0.797-0.865)	0.728	0.835	0.556	0.806 (0.765-0.847)	0.718	0.831	0.594
Location model	0.826 (0.805-0.847)	0.803	0.882	0.658	0.807 (0.771-0.842)	0.788	0.876	0.634	0.842 (0.804-0.879)	0.809	0.895	0.649
Loc-Cli model	0.923 (0.909-0.938)	0.887	0.936	0.766	0.909 (0.883-0.935)	0.864	0.924	0.752	0.912 (0.883-0.941)	0.893	0.939	0.774
**Prognosis**												
Clinical model	0.799 (0.766-0.832)	0.716	0.670	0.800	0.772 (0.717 -0.821)	0.647	0.528	0.830	0.762 (0.666-0.795)	0.758	0.848	0.656
Location model	0.785 (0.753 -0.815)	0.705	0.652	0.800	0.725 (0.668 -0.778)	0.647	0.521	0.840	0.777 (0.708-0.830)	0.773	0.838	0.699
Radiomics model	0.839 (0.810 -0.866)	0.769	0.777	0.756	0.831 (0.780 -0.873)	0.751	0.755	0.745	0.840 (0.782-0.889)	0.783	0.829	0.731
Loc-Cli-Rad model	0.895 (0.869 -0.916)	0.855	0.888	0.796	0.874 (0.828 -0.911)	0.829	0.865	0.774	0.850 (0.790-0.895)	0.879	0.905	0.849

AUC= Area under the curve; Loc-Cli model = Location- Clinical model; Loc-Cli-Rad model= Location- Clinical- Radiomics model

Supplementary Table 5. Delong analysis of AUC values of models

	Dateset1			Dateset2			Dateset3		
**Delong test-etiology**									
	AUC (Cli)	AUC (Loc-Cli)	p value	AUC (Cli)	AUC (Loc-Cli)	p value	AUC (Cli)	AUC (Loc-Cli)	p value
**Etiology**	0.848	0.923	< 0.0001	0.831	0.909	0.0001	0.806	0.912	0.0002
**Delong test-prognosis**									
	AUC (Cli)	AUC (Loc-Cli-Rad)	p value	AUC (Cli)	AUC (Loc-Cli-Rad)	p value	AUC (Cli)	AUC (Loc-Cli-Rad)	p value
**Prognosis**	0.799	0.895	< 0.0001	0.772	0.874	0.003	0. 762	0.850	0.004

Cli = clinical; Loc-Cli=location-clinical; Loc-Cli-Rad= location-clinical-radiomics; AUC= AUC= Area under the curve.
